# Nanotechnology Applications of Flavonoids for Viral Diseases

**DOI:** 10.3390/pharmaceutics13111895

**Published:** 2021-11-08

**Authors:** Khoshnur Jannat, Alok K. Paul, Tohmina A. Bondhon, Anamul Hasan, Muhammad Nawaz, Rownak Jahan, Tooba Mahboob, Veeranoot Nissapatorn, Polrat Wilairatana, Maria de Lourdes Pereira, Mohammed Rahmatullah

**Affiliations:** 1 Department of Biotechnology & Genetic Engineering, University of Development Alternative, Lalmatia, Dhaka 1207, Bangladesh; jannat.koli.22@gmail.com (K.J.); alok.paul@utas.edu.au (A.K.P.); afrozebondhon@gmail.com (T.A.B.); anamulhasanoris@gmail.com (A.H.); rownak86@hotmail.com (R.J.); 2 School of Pharmacy and Pharmacology, University of Tasmania, Hobart, TAS 7001, Australia; 3 Department of Nano-Medicine Research, Institute for Research and Medical Consultations (IRMC), Imam Abdulrahman Bin Faisal University, Dammam 31441, Saudi Arabia; nawwaz@gmail.com; 4 School of Allied Health Sciences, World Union for Herbal Drug Discovery (WUHeDD) and Research Excellence Center for Innovation and Health Products (RECIHP), Walailak University, Nakhon Si Thammarat 80160, Thailand; tooba666@hotmail.com (T.M.); nissapat@gmail.com (V.N.); 5 Department of Clinical Tropical Medicine, Faculty of Tropical Medicine, Mahidol University, Bangkok 10400, Thailand; 6 CICECO-Aveiro Institute of Materials & Department of Medical Sciences, University of Aveiro, 3810-193 Aveiro, Portugal; mlourdespereira@ua.pt

**Keywords:** flavonoids, anti-viral, nanotechnology, bioavailability, drug delivery

## Abstract

Recent years have witnessed the emergence of several viral diseases, including various zoonotic diseases such as the current pandemic caused by the Severe Acute Respiratory Syndrome Coronavirus 2 (SARS-CoV-2). Other viruses, which possess pandemic-causing potential include avian flu, Ebola, dengue, Zika, and Nipah virus, as well as the re-emergence of SARS (Severe Acute Respiratory Syndrome) and MERS (Middle East Respiratory Syndrome) coronaviruses. Notably, effective drugs or vaccines against these viruses are still to be discovered. All the newly approved vaccines against the SARS-CoV-2-induced disease COVID-19 possess real-time possibility of becoming obsolete because of the development of ‘variants of concern’. Flavonoids are being increasingly recognized as prophylactic and therapeutic agents against emerging and old viral diseases. Around 10,000 natural flavonoid compounds have been identified, being phytochemicals, all plant-based. Flavonoids have been reported to have lesser side effects than conventional anti-viral agents and are effective against more viral diseases than currently used anti-virals. Despite their abundance in plants, which are a part of human diet, flavonoids have the problem of low bioavailability. Various attempts are in progress to increase the bioavailability of flavonoids, one of the promising fields being nanotechnology. This review is a narrative of some anti-viral dietary flavonoids, their bioavailability, and various means with an emphasis on the nanotechnology system(s) being experimented with to deliver anti-viral flavonoids, whose systems show potential in the efficient delivery of flavonoids, resulting in increased bioavailability.

## 1. Introduction

At the moment, we are undergoing a global pandemic caused by a zoonotic Coronavirus, Severe Acute Respiratory Syndrome Coronavirus 2 or SARS-CoV-2, which is responsible for the disease known as COVID-19 (for Coronavirus disease 2019), because it emerged at the tail end of the year 2019 [1]. A zoonotic virus is a virus that has been transmitted from an animal to a human, sometimes with the involvement of a secondary human host. SARS-CoV-2 is believed to have come from bats through a yet unidentified secondary host, mostly hypothesized to be pangolins, but also hypothesized as ferrets, minks, snakes, and turtles by different groups of researchers [2].

Two other coronaviruses with pandemic potential, namely Severe Acute Respiratory Syndrome (SARS) and Middle East Respiratory Syndrome (MERS) emerged before SARS-CoV-2. SARS was first reported in Guangdong Province in China in November 2002 and lasted until 2004, with more than 8000 reported cases; the virus originated from bats, with civet cats being the secondary hosts responsible for transmission to humans. MERS was first observed in 2012 in Saudi Arabia and infected 2494 confirmed cases; once again, bats were the primary hosts, with dromedary camels as secondary hosts [3]. In comparison, the SARS-CoV-2 pandemic shows very little signs of abating and as of 3 August 2021, it was reported to have caused 199,644,866 infections, resulting in 4,250,237 deaths in practically every country in the world [4].

A number of zoonotic and non-zoonotic viruses have the potential to create pandemics. These viruses include human Metapneumo virus (hMPV), Respiratory syncytial virus (RSV), severe fever with thrombocytopenia syndrome virus (SFTSV), Hanta virus (HTNV), Sin Nombre virus (SNV), avian Influenza A strain (H7N9), Ebola virus, Zika virus, Nipah virus, Hepatitis viruses, West Nile fever virus, Lujo virus, Lassa virus (LASV), Dengue virus (DENV), and Chikungunya virus (CHIKV), to name only a few [5,6,7,8]. One point to note is that these viruses are, on top of other ‘common’ viruses like Human immunodeficiency virus (HIV) and human Papilloma virus (HPV), currently infecting millions of people, but not attracting a great deal of general attention, probably because they have been around for some time and at least some forms of treatment are now available for them [9]. For instance, Acquired Immunodeficiency Syndrome (AIDS), caused by HIV, was first recognized as a new disease in 1981 and has since been responsible for at least 60 million infections and at least 25 million deaths [10].

## 2. Anti-Viral Drugs and Vaccines

### 2.1. Viral Diseases and Conventional Anti-Viral Drugs

The foremost thing that can be said of conventional anti-viral drugs is that most viruses and particularly emerging zoonotic viruses do not have suitable drugs or vaccines to effectively fight the various virus-induced diseases. According to a review [11], existing antiviral drugs can be generalized into two classes: drugs targeting the virus itself and drugs targeting host cell factors. A virus needs attachment and subsequent entry into host cells and as such, inhibitors targeting the virus can be compounds that prevent a virus from attaching to the host cell receptor and thus gaining entry into the host cell. Other inhibitors can be viral proteases and other factors involved in viral replication within the host cell.

In 1963, the United States Food and Drug Administration (USFDA) first approved an anti-viral drug, idoxuridine, to treat Herpes simplex virus (HSV) infections.

Since then, zidovudine, didanosine, zalcitabin, and 49 other drugs have been approved by the USFDA for the treatment of HIV; elbasvir, sofosbuvir, and 16 other drugs for treatment of Hepatitis C virus (HCV); 8 drugs for the treatment of Hepatitis B virus (HBV), including lamivudine and entecavir; oseltamivir, favipiravir, and 7 other drugs against Influenza virus; pensiclovir, famciclovir, and 9 other drugs against Herpes simplex virus (HSV), and 19 other compounds for the treatment of human Papilloma virus (HPV), Respiratory syncytial virus (RSV), human Cytomegalovirus (HCMV), and Varicella-zoster virus (VZV) [12].

There are currently no FDA-approved drugs against Ebola virus (a filovirus), although remdesivir has been found to be active against several ‘variants’ of the virus [13]. The absence of effective specific virus-targeting drugs also applies to ‘emerging’ viruses such as another filovirus Marburg, henipaviruses (Nipah, Hendra), Lassa virus, Lujo virus, Hanta virus, Rift valley fever virus, tick-borne encephalitis viruses, arboviruses such as Dengue, Chikungunya, Japanese encephalitis, West Nile, yellow fever and Zika virus, South American hemorrhagic fever viruses, as well as SARS, MERS, and SARS-CoV-2 (note that this is not a complete list) [14,15]. Treatments for these viruses are mainly symptomatic or with repurposed drugs, which may act in only a percentage of all cases. For instance, several drugs, which were chosen for the treatment of COVID-19 based on their previous potential efficacies against other viral diseases, did not demonstrate much promise when used against SARS-CoV-2. Some of these drugs include favipiravir (approved for the Influenza virus in Japan) or the lopinavir-ritonavir combination (HIV-1 protease inhibitor) [16,17], or even the most widely used drug against COVID-19, namely remdesivir, which was developed to treat the Ebola virus and showed broad-spectrum anti-viral activity in animal models and tissue culture [18].

### 2.2. Viral Diseases and Vaccines

If drugs are taken out of account, another line of defense is the use of vaccines against a virus. Vaccines have proven to be a success story in recent decades, where the use of vaccines was the primary reason for eliminating smallpox from the world and is on the verge of eliminating poliomyelitis. Other viral diseases in children and adults, such as measles, mumps, yellow fever, chicken pox, rubella, rabies, and Hepatitis B have been brought under control. Lately, vaccines have been developed against human Papilloma virus (one of the main causative agents of cervical cancer) and Rotavirus (the causative agent of diarrhea, particularly in children under 5 years of age) [19].

The Center for Disease Control (CDC) in the USA lists the following diseases where vaccines are used for preventive purposes. Diseases include chicken pox, diphtheria, flu (Influenza), Hepatitis A, Hepatitis B, *Haemophilus influenzae* type b (Hib), human Papilloma virus, measles, meningococcal, mumps, pneumococcal, polio, Rotavirus, rubella, shingles, tetanus, whooping cough (pertussis), anthrax, Japanese encephalitis, rabies, smallpox (no longer necessary), tuberculosis, typhoid fever, and yellow fever [20]. On the other hand, it has been difficult to produce vaccines against the newly emerging zoonotic viral diseases.

Recent research has enabled scientists to come out with two effective Ebola vaccines that have received regulatory approval, namely r-VSV-ZEBOV (Merck) and Ad26.ZEBOV/MVA-BN-Filo (Janssen Vaccines and Prevention). Despite the success of the research, the implementation of an effective vaccine program has been complicated by several factors, such as whether it is economically feasible to vaccinate over a billion people in Ebola-prone areas, with thus far 30 outbreaks involving fewer than 40,000 cases and the remoteness of the population [21].

Thus, one of the problems in developing vaccines against newly emerging zoonotic viruses, as well as other viruses, is the problem of cost-acceptability. It takes several years and billions of dollars to develop a new drug or vaccine and it is simply not worthwhile for pharmaceutical companies to embark on such a path when potential recipients are few in the absence of a major outbreak or pandemic. Moreover. by the time a pandemic really occurs as in the case of SARS-CoV-2, despite the best efforts of scientists, hundreds of thousands of lives have been lost before the discovery of vaccines against this virus, whose vaccines are still administered under ‘emergency use approval’, because adequate time for thorough testing was and is simply not at hand. This occurred because it was previously thought that with the ‘disappearance’ of SARS and MERS, it was not necessary to invest more time and money into full-scale Coronavirus vaccine research and so all research practically stopped. Despite the rapid development of COVID-19 vaccines, the first ‘emergency use approval’ (EUA) was issued by the USFDA on 11 December 2020 [22], which is nearly a year after the disease started. The problems associated with viral vaccines will be more elaborated in this section with reference to SARS-CoV-2.

The situation with SARS-CoV-2 highlighted other problems regarding the development and subsequent administration of vaccines. Conventional vaccines use attenuated or inactivated viruses. Apart from inactivated virus (Sinovac), the other vaccines for COVID-19 that have gained ‘emergency administration approval’ are mRNA-based (PfizerBioNTech and Moderna) or viral-vector-based (AstraZeneca-Oxford and Janssen Pharmaceutical) [23]. The emergence of ‘variants of concern’, such as the delta variant of SARS-CoV-2 (which appears to have become the main cause of COVID-19 resurgence) has raised concerns about the efficacy of current vaccines in meeting this new mutant and possibly other mutants that may occur in the future [24]. Concerns have been raised in the general public (public hesitancy) towards the acceptance of COVID-19 vaccines, partly fueled by loose talk from a section of the intelligentsia about the anticipated dangers of vaccines to long-term public health because of the rapidity of the vaccine development and the rapid clearance given to the administration of vaccines, which have been developed through what are considered to be new methods [25]. A recent review found that while the acceptance rate for the COVID-19 vaccine is as high as 97.0% in Ecuador, it is 56.9% in the USA and only 23.6% in Kuwait [26].

A high vaccination rate (around 70% or above of the population) has been recommended by experts such as Dr. Anthony Fauci, Director of the National Institute of Allergy and Infectious Diseases to develop ‘herd immunity’ and so prevent COVID-19 from further spread [27]. This high rate of vaccination around the world has not happened so far in practice, not only because of vaccine reluctance, but also because of a number of factors. The current world population is around 7.9 billion [28]. To achieve a vaccination rate of 70%, it requires vaccinating at least 5.5 billion people. Since most of the current vaccines need two doses for effective immunization (PfizerBioNTech now calls for three doses to protect against the delta variant of SARS-CoV-2), this requires more than 11 billion doses, assuming loss during storage or transit. Moreover, the world population is not concentrated in one place or in several large centers, but widely distributed, quite often in rather inaccessible and remote regions. As a result, effective dissemination and deployment of vaccines poses a real problem.

The cost of vaccines is a matter of concern for both the population and the governments of low-income countries (LICs) and low-middle-income countries (LMICs). This factor has resulted in the appropriation of vaccines by richer nations at more than the necessary doses at the expense of LICs and LMICs, a condition frequently referred to now as ‘vaccine apartheid’.

Other factors of concern are unexpected delays in supply chains, selling spurious vaccines, and the storage of vaccines after reaching their destinations (for instance, the PfizerBioNTech vaccine needs ultra-cold temperatures of around −70 °C for storage and should not be repeatedly freeze-thawed). About 20% of the world’s poorest countries do not have adequate cold chain capacities. Global shortages of glass vials and syringes due to the extra demand for vaccinations are other problems that have manifested following the initiation of vaccine drive [29].

### 2.3. Consideration of Alternative Anti-Viral Treatments

To summarize, a number of concerns have been raised with current anti-viral drugs and vaccines. Vaccines are not cost-effective to research and produce unless there is a pandemic and by that time, the pandemic can result in loss of huge numbers of lives. If the case fatal rate (CFR) among the three major Coronaviruses is compared, SARS, MERS, and SARS-CoV-2 CFRs were, respectively, 9.6, 34.3 and 4.4% [30]. A CFR of 90% has been estimated for Ebola virus cases in Africa [31]. The CFR of Lassa fever in Nigeria was the highest in 2017 (26.5%) with CFRs of 23.7%, 19.6%, and 13.4% in 2018, 2019 and 2020, respectively [32]. In these days of globalization and continuous movement of people between various countries around the world, it is far easier to have a high mortality number from a pandemic with high CFR before scientists can come up with an appropriate solution. This raises the question of whether vaccine production methodology should be optimized against emerging viruses to enable rapid vaccine production in time of need and if so, who is going to bear the enormous costs involved in vaccine research? Moreover, what happens if a virus re-emerges in a mutant form?

Some of these anti-virals, for instance, in the case of SARS-CoV-2, may work in selective cases or situations and then still can be controversial in their use because of conflicting reports. For instance, remdesivir is advised by the USFDA to be administered in a hospital or health-care setting. Liver and renal function tests should be done before and during administration of the drug [33]. On the other hand, a study conducted in China with 237 patients (158 in the remdesivir group and 79 in the placebo control group) showed that remdesivir did not reduce the time for clinical improvement. Overall, the study concluded that remdesivir had no clinical benefits because the drug did not reduce mortality and viral clearance time in patients with severe COVID-19 compared to the placebo control group [34]. A recent review, on the other hand, concluded that remdesivir can reduce viral load and inhibit SARS-CoV-2 replication, but needs further efficacy assessments [35]. Added to these conflicting reports is the affordability. The cost of remdesivir in a LMIC such Bangladesh (manufactured by a Bangladeshi pharmaceutical company) is around 5000–6000 Bangladeshi Taka (BDT) per vial (USD 58–71 per vial) and a patient may need 5–11 vials of the drug, which is administered by intravenous infusion [36]. To be noted is that the per capita income in Bangladesh has been quoted at USD 2227 for the 2020–2021 fiscal year [37].

Xofluza (generic name—baloxavir marboxil) was approved by the USFDA in 2018 for the treatment of Influenza virus. Common adverse drug reactions include diarrhea, bronchitis, nasopharyngitis, headache, and nausea [38]. Moreover, although the medication must be taken in a single dose, prices are quoted at around USD 154.50, making the medication essentially unaffordable for low-income people without any health insurance, as is the case with many LICs and LMICs.

Prevymis (generic name—Letermovir) was approved by the USFDA in 2017 for the treatment of human Cytomegalovirus (HCMV). The most common adverse reactions to this drug have been mentioned as nausea, diarrhea, and vomiting by the European Medicines Agency [39]. The cost of the Prevymis oral tablet 240 mg is around USD 3309 for a supply of 14 tablets; the recommended dosage for this drug is 480 mg administered orally or intravenously once a day for several days as recommended by a physician. 

Taken together, conventional synthetic anti-viral drugs not only have adverse effects, but their prices are beyond the range of low-income people. It is ironic but true that, for instance, the price of only 14 tablets of 240 mg Prevymis is much more than the average per capita income of most LICs and LMICs. To make anti-viral drugs more affordable and, particularly against emerging viruses, more available, it is time to look at new sources of anti-viral drugs, the most obvious and most common source of drugs traditionally being plants.

## 3. Plants as Sources for New Anti-Viral Drugs

Terrestrial plants have a long history of being used by humans for disease treatment. The first known written record as found in the clay tablets of Mesopotamia (circa 2600 BC) mentions medicinal uses of licorice from *Glycyrrhiza glabra* L. (Fabaceae), myrrh from *Commiphora myrrha* (Nees) Engl. (Burseraceae), and poppy capsule latex from *Papaver somniferum* L. (Papaveraceae). These plants or their isolated active ingredients are still used in conventional and traditional medicines at present [40]. Plants produce phytochemicals known as secondary metabolites; the diverse pharmacological activities of these secondary metabolites are utilized by scientists to produce new drugs or scientists use their basic structure as a scaffold to produce synthetic drugs. The various secondary metabolites produced by plants include alkaloids, flavonoids, tannins, saponins, sterols, limonoids, polyphenols, glucosinolates, terpenes and other groups of bioactive compounds; they are produced by plants for repelling herbivores and insects, as well as for undergoing stress conditions like drought. Many of these secondary metabolites are being used as conventional drugs; others have the potential of being future drugs. Early drugs of plant origin include cocaine, codeine, digitoxin, and quinine; some modern drugs originated from plants include artemisinin, ajmaline, allicin, andrographolide, berberine, curcumin, reserpine, taxol, vincristine, and vinblastine [41].

One estimate puts the number of flowering plant species in the world as exceeding 352,000 [42]. Since all these plants produce varying amounts and diverse types of phytochemicals, this opens up a vast opportunity for scientists to discover novel drugs from plants for old and emerging diseases like the viral diseases. A search in PubMed Central with the terms <alkaloids> and <viral diseases> resulted in 10,083 hits. Among the anti-viral alkaloids described in a recent review [43], the alkaloid lycorine from the plant *Lycoris radiata* L. (Amaryllidaceae) reportedly has a broad anti-viral activity spectrum, including DV [44], ZV [45], Poliovirus [46], HCV [47], Enterovirus 71 (EV-71) and Coxsackievirus A16 [48,49], avian Influenza H5N1 virus [50], HSV1 [51], and Bunyaviruses and Rift Valley fever virus (RVFV) [52]. Notably, anti-SARS-CoV and SARS-CoV-2 activities have also been reported for lycorine [53,54].

The above represents only one example of the potential of alkaloids against viral diseases. A search in PubMed Central with the terms <flavonoids> and <viral diseases> resulted in 11,811 hits, demonstrating that flavonoids have greater potential against various viruses as a potential agent for prophylaxis and therapy. The rest of the review will concentrate on flavonoids, their anti-viral activities, and the efficient mode of delivery to have a greater impact against various viruses, including SARS-CoV-2. 

### 3.1. Flavonoids—Classification, Structure, and Anti-Viral Activities

**Figure 1:**
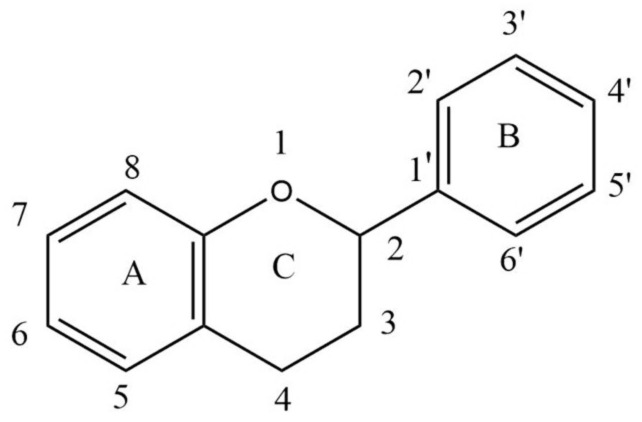
Basic structure of a flavonoid.

Flavonoids are secondary metabolites present in plants as derivatives of 2-phenyl-benzo-γ-pyrone. There are two benzene rings in flavonoids (usually designated as A and B), which are connected by an oxygen containing pyrene ring (C) in a C6-C3-C6 system. The basic structure of a flavonoid is shown in Figure 1.

**Figure 2:**
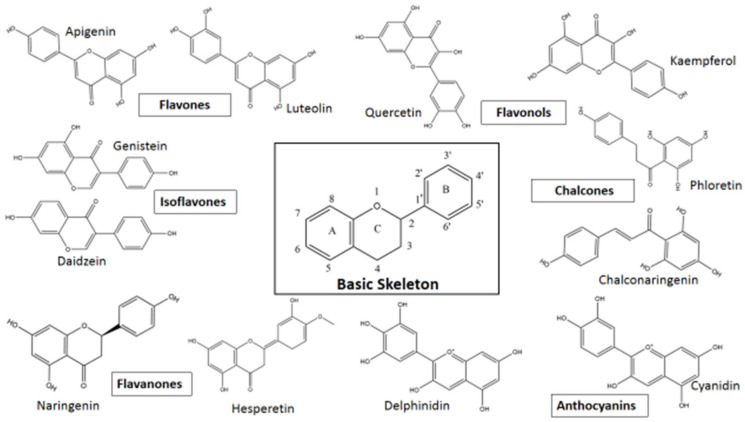
Flavonoid sub-groups with some representative examples.

Based on variations in the basic structure of a flavonoid, as depicted in Figure 1, flavonoid compounds can be sub-grouped into flavanols, flavanones, flavonols, isoflavones, flavones, and anthocyanins; some other groups can also be included, such as bioflavonoids, prenyl-flavonoids, flavonolignans, glycosidic ester flavonoids, chalcones, and proanthocyanins [55,56]. Some flavonoid sub-groups are shown in Figure 2. Figure 2 does not show all sub-groups of flavonoids but indicates the common and important groups. A representative example of each sub-group shall be discussed with respect to its anti-viral activity, followed by giving a tabular presentation of a number of flavonoids along with their various antiviral activities. In a tabular presentation, more importance shall be given to dietary flavonoids. However, we point out that this tabular presentation, because of the sheer number of flavonoids present (both dietary and non-dietary), is not a comprehensive list, but rather a list of flavonoids chosen somewhat at random, which the existing literature suggests to have a comparatively broader range of anti-viral activities. This is an important issue; SARS, MERS, CARS-CoV-2, Ebola—all these viral diseases suddenly appeared and caught human beings totally unprepared. In these types of diseases, broad spectrum anti-viral compounds may have greater potential to tackle the new viral disease. 

Flavonoids can inhibit viruses in various ways. For instance, Coronaviruses need to enter the human host cell through binding of its spike glycoprotein (S) with a receptor (human angiotensin converting enzyme 2 or hACE-2) and with the help of other host factors, such as transmembrane serine protease 2 (TMPRSS2) and endosomal cysteine proteases cathepsin B (Cat B) and cathepsin l (Cat l), gain entry into the host cell. Viral RNA is then freed from its protein coat and released followed by immediate translation of two large open reading frames, ORF1a and ORF1b. The resulting polyproteins pp1a and pp1ab are processed into individual nonstructural proteins (nsps) by two viral proteases Mpro and PLpro, with nsps playing a vital role in viral replication [57,58]. Flavonoids can then act as potential therapeutics by inhibiting various targets in this whole process such as binding to S protein receptor binding domain (RBD), hACE2, TMPRSS2, CatB, CatL, as well as two viral proteases, namely chymotrypsin-like protease (Mpro) and papain-like protease (PLpro), which in turn suggests that flavonoids have the potential to inhibit virus binding, entry, and replication.

In this section, a few examples of anti-viral actions of various flavonoids will be discussed. We take this opportunity to mention that the present review is not a systematic review in the sense of systematically categorizing and discussing the various thematic issues discussed in various sections of the manuscript, but rather an overall description of the ‘state of the current perspective’ followed at the end with a discussion of future directions on both the use of flavonoids as anti-viral drugs and their efficient mode of delivery to the body, because flavonoids, as will be discussed in a later section, are plentiful in the human diet but of limited availability because of their poor absorption.

As reviewed by Tapas et al. and Ahmad et al. [59,60], flavone compounds include apigenin, diosmin, luteolin, chrysin, vitexin, orientin, and isoorientin; apigenin (4′,5,7-trihydroxy-flavone) reportedly inhibits foot-and-mouth disease virus (FMDV) [61], inhibition of Enterovirus-A71 infection by apigenin has been shown to occur via suppression of the internal virus entry site of the Enterovirus [62]; the compound has been shown to suppress the activity of the immediate-early (IE) gene Zta and Rta promoters of the Epstein-Barr virus (EBV), suggesting that apigenin can block initiation of the EBV lytic cycle [63]; the compound extracted from *Mosla scabra* (Thunb.) C.Y. Wu and H.W. Li (Lamiaceae) demonstrated anti-viral activity against Influenza viruses [64]; the compound inhibits Hepatitis C virus (HCV) by decreasing MicroRNA122 (miR122), which is expressed in the liver and essential for the stability and propagation of HCV RNA [65].

Flavonols are flavonoids with a ketone group. Flavonols have an –OH group at the 3-position of the C-ring; the hydroxyl group may be glycosylated [66]. Quercetin and kaempferol are two important members of the flavonol sub-group of flavonoids. Other notable members include galangin, fisetin, myricetin, and vitexicarpin. Myricetin (3,5,7-trihydroxy-2-(3,4,5-trihydroxyphenyl)-4-benzo-pyrone) reportedly inhibited African swine fever virus (ASFV) protease with an IC_50_ value of 8.4 μM. Myricitrin, a derivative of myricetin with a rhamnoside moiety, also inhibited ASFV [67]. The highly immunogenic glycoprotein d (gd) of Herpes simplex virus 2 (HSV-2) plays an important part in viral entry into host cells. Myricetin directly interacts with the viral gd protein to block virus adsorption and membrane fusion to host cells. Furthermore, myricetin also down-regulates the host EGFR/PI3K/Akt (epidermal growth factor receptor/phosphoinositide 3-kinase/Akt or protein kinase B) signaling pathway, which inhibits viral infection and replication [68].

A study was conducted on the effect of elderberry extract on Influenza H1N1 virus in MDCK (Madin–Darby canine kidney) cells. Among the constituents of the extract, (±) dihydromyricetin was found to potently inhibit the virus with an IC_50_ value of 8.7 μM [69]. Myricetin also inhibited Zika virus replication through nearly complete inhibition of viral RNA production [70]. Myricetin and dihydromyricetin can reportedly modulate the infectivity of Enterovirus A71 (EV71), but the mechanism of inhibition has not been elucidated [71]. Viral inhibition of Hepatitis B virus (HBV) has been noted in vitro with myricetin rhamnoside (MyrG) and myricetin-3α-*O*-rhamnosyl (1→6)-α-galactoside (MyrGG) obtained from *Marcetia taxifolia* DC. (Melastomataceae) [72]. The in vitro anti-HBV activity has also been described for myricetin-3-*O*-rhamnoside isolated from *Guiera senegalensis* J.F. Gmel. (Combretaceae) leaves [73]. Strong inhibitory activity by myricetin obtained from *Marcetia taxifolia* and *Dioscorea bulbifera* L. (Dioscoreaceae) against HIV-1 reverse transcriptase (RT) and HIV-1 integrase has been reported [74,75,76]. The glycosylated moiety of myricetin possibly favors internalization within the cell and enhances anti-HIV-1 activity [76]. 

Myricitrin (myricetin-3-*O*-rhamnoside) present in *Newtonia hildebrandtii* (Vatke) Torre (Fabaceae) and *Newtonia buchananii* (Baker) G.C.C. Gilbert and Boutique (Fabaceae) leaf extracts have been shown to have high anti-viral efficacy against influenza A virus (IAV) PR8/34/H1N1 as a model organism [77]. Myricitrin appears to exert its anti-viral effects by inhibiting viral attachment and entry [77]. Myricetin has been reported to inhibit ATPase activity of the SARS helicase nsP13 by more than 90% at a concentration of 10 μM, while myricitrin showed around 20% inhibition. In vitro analysis gave an IC_50_ value of myricetin against ATPase of 2.71 ± 0.19 μM [78].

In silico studies show that myricetin can bind to SARS-CoV-2 Mpro with the chromone ring of the compound by interacting with His41 (one of the catalytic dyad amino acid residues of Mpro) through π–π stacking, as well as the formation of hydrogen bonds between 3′-, 4′- and 7-hydroxyl of myricetin and Phe140, Glu166, and Asp187 of Mpro [79]. Myricetin also showed an anti-inflammatory effect on bleomycin-induced pulmonary inflammation; infiltration of inflammatory cells and secretion of inflammatory cytokines like interleukin-6 (IL-6), IL-1α, tumor necrosis factor-α (TNF-α), and interferon-γ (IFN-γ) were inhibited [79]. Two flavonoids, dihydroxy-6′-methoxy-3′,5′-dimethylchalcone and myricetin-3′,5′-dimethyl ether 3-*O*-β-d-galactopyranoside isolated from *Cleistocalyx operculatus* (Roxb.) Merr. and L.M. Perry (Myrtaceae) inhibited the replication of Influenza virus H1N1 in MDCK cells [80]. In silico studies have shown that myricitrin and myricetin-3-*O*-rutinoside have strong docking scores with the active site of Mpro in SARS-CoV-2 [81]. Myricetin showed in silico studies good binding affinities for both Mpro and endoribonuclease of SARS-CoV-2 [82]. Myricetin showed an IC_50_ value of 43 ± 1 μM when tested in vitro assays of Mpro [83].

Chalcones ((E)-1,3-diphenylpropen-1-ones) consist of two aromatic rings (A and B) attached by an α,β-unsaturated carbonyl system; chalcones and flavanones are isomeric structures belonging to the flavonoid family [84]. More than 600 naturally occurring chalcones have been identified, mostly in plants of the Asteraceae, Fabaceae, and Moraceae family [85]. A naturally occurring chalcone, phloretin, has been reported to significantly reduce Zika virus (ZIKV) titres in infected Vero cells. Phloretin was active against two ZIKV strains, the African strain MR766 and the Puerto Rican strain PRVABC59, the EC_50_ concentrations being 22.85 and 9.31 μM, respectively. The inhibitory action of phloretin was attributed to the inhibition of glucose uptake by cells, thus limiting viral replication [86].

Anthocyanins are a sub-group of flavonoids that render various colors such as red, blue, pink, or purple to flowers, vegetables, and fruits. They have the flavylium (2-phenylchromenylium) ion, a positive charge at the oxygen atom of the C-ring of the flavonoid basic structure [87]. From a structural point of view, anthocyanins are anthocyanidins which have been modified by acyl acids (acylated) or sugars (glycosylated). The number of hydroxyl groups on the B ring determines the extent of blue color of anthocyanins; methylation leads to a red color [88]. Common anthocyanidins in plants are cyanidin, delphinidin, pelargonidin, peonidin, malvidin, and petunidin, with distribution levels in fruits and vegetables at 50%, 12%, 12%, 12%, 7%, and 7%, respectively [89].

Delphinidin (3,5,7-trihydroxy-2-(3,4,5-trihydroxyphenyl)-1λ-4-chromen-1-ylium) has been reported to inhibit viruses of the *Flavivirus* genus such as the West Nile virus (WNV), Zika virus (ZIKV), and Dengue virus (DENV). The compound was found to affect the viral attachment and entry into host cells. A virucidal effect was also observed; delphinidin further reduced the infectivity of ZIKV and DENV [90]. In experimental studies, it was found that delphinidin attaches directly to viral particles and impairs viral (HCV) attachment to the host cell surface, which is a new mechanism of action for inhibiting viral entry. It was also active against the virus in primary human hepatocytes. The IC_50_ of delphinidin in HCV-infected Huh-7 cells was 3.7 ± 0.8 μM [91]. 

The anti-human Influenza A activity of *Hibiscus sabdariffa* L. (sorrel) has been attributed to a number of phytochemical constituents, including delphinidin-3-*O*-sambubioside [92]. In silico studies have shown that delphinidin-3,5-diglucoside interacts via hydrogen bonding with Gly143, His163, His164, Glu166, Gln189, Thr190, and Gln192 and π–π interaction with His41 of the main protease Mpro of SARS-CoV-2, which suggests that the compound can be useful in inhibiting viral replication through inhibition of Mpro [93]. The compound can also reportedly bind to ACE-2, the human receptor for SARS-CoV-2 spike protein [94]. A number of flavonoid compounds were screened by molecular docking techniques for their binding ability against main protease, RNA-dependent RNA polymerase, and spike proteins of SARS-CoV-2 using AutoDock 4.1. Among the various flavonoids tested, Delphinidin 3-*O*-β-d-glucoside 5-*O*-(6-coumaroyl-β-d-glucoside) was identified as a potent inhibitor of all three protein targets of SARS-CoV-2 [95].

Flavanones, previously regarded as a minor group of flavonoids, are now considered a major group with about 350 flavanone aglycones and 100 flavanone glycosides identified from natural sources [96]. One of the main flavanones is naringenin ((*S*)-5,7-dihydroxy-2-(4-hydroxyphenyl) chroman-4-one), which is a colorless and flavorless flavanone. It is the main flavanone in grapefruit. Although COVID-19 pneumonia is different from bronchopneumonia, the latter being a bacterial infection [97], it is interesting to note that naringenin reportedly can alleviate bronchial pneumonia in children [98].

Naringenin was tested against Chikungunya virus (CHIKV) replicon transfected into BHK cells (baby hamster kidney cells). The replicon contained virus replicase proteins with puromycin acetyl transferase, EGFP (enhanced green fluorescent protein), and Renilla luciferase (Rluc) marker genes. It was found that naringenin suppresses activities of Rluc and EGFP marker genes expressed by the CHIKV replicon. The anti-alphaviral activity of naringenin was also confirmed against Semliki forest virus (SFV). Naringenin further reduced SFV and Sindbis virus-induced cytopathic effect and inhibited SFV virion production [99]. Naringenin showed potential as a CHIKV inhibitor in silico studies by demonstrating good binding affinity to nonstructural protein 2 (nsP3), which is considered an important protein in the intracellular replication of the virus [100].

Naringenin, when added to Huh7.5 cells before and after cells were infected with Dengue virus (DENV) serotypes 1–4, reduced the number of DENV-infected cells. Furthermore, the compound inhibited replication of the virus in Huh7.5 cells; this inhibition of replication was confirmed in two replicons of the virus, namely DENV-1 and DENV-3 replicons, which contain non-structural viral proteins that can enable RNA replication and translation without viable viral particle assembly. 

Naringenin was further found to inhibit DENV infection in human monocytes [101]. In another study, naringenin exhibited virucidal activity against DENV serotype 2 with an IC_50_ value of 52.64 μg/mL [102]; the anti-adsorption effects of naringin (naringenin is the precursor of naringin) has been reported for Vero cells against the DENV-2 New Guinea C strain with IC_50_ of 162.8 μg/mL [103].

Hepatitis B virus (HBV) protein X (HBx) induces hepatic steatosis. Naringenin has been reported to reduce HBx-induced expression of hepatic adipogenic and lipogenic genes and so prevent HBx-induced hepatic steatosis. The underlying mechanism includes suppression of HBx-induced gene expressions, including decreases in the transcriptional activity of sterol regulatory element-binding protein 1c (SREBP1c), liver X receptor α (LXRα), and peroxisome proliferator-activated receptor γ (PPARγ) in HBx-trangenic mice and HBx-transfected HepG2 cells [104].

Production of the Hepatitis C virus in cells was found to be blocked by naringenin. The compound blocked the intracellular assembly of virus particles prior to viral egress from the cell(s). It was shown that this anti-viral effect was at least partially mediated by PPARα leading to reduced production of very low-density lipoproteins (VLDL), which is necessary for viral assembly [105]. In HCV infections, the virus is secreted by infected cells in a Golgi-dependent pathway while bound to VLDL. Silencing ApoB mRNA can lead to a 70% reduction in the secretion of ApoB, HCV core protein, and HCV RNA. Naringenin can inhibit ApoB secretion, which is achieved by inhibiting the activity of the microsomal triglyceride transfer protein (MTP) as well as the transcription of 3-hydroxy-3-methyl-glutaryl-coenzyme reductase (HMGR) and acyl-coenzyme A: cholesterol acyltransferase 2 (ACAT2) [106].

The capsid core protein of HCV plays the key role in the assembly and packaging of the HCV RNA genome. In silico studies indicate that naringenin can inhibit the core capsid protein of HCV-genotype 3 (G3) (Q68867) and its subtypes 3b (Q68861) and 3g (Q68865) from north India [107]. It has been reported that naringenin can inhibit Zika virus (ZIKV) infection in human A549 cells in a concentration-dependent manner. This antiviral activity was also observed with ZIKV-infected primary human monocyte-derived dendritic cells. 

This anti-viral activity of the compound was observed following treatment of infected cells after infection, suggesting that the compound targets viral assembly or viral replication. Molecular docking analysis also showed that naringin can interact with the protease domain of NS2B-NS3 protein of ZIKV [108]. The NS2B-NS3 cofactor-protease assembly is necessary for cleavage of the ZIKV polyprotein precursor and generation of fully functional ZIKV proteins [109].

Naringenin has the potential to be an effective therapeutic against SARS-CoV-2. A recent review concluded from an appraisal of the evidence thus far collected that naringenin can act against SARS-CoV-2 and COVID-19 in at least four ways. The compound can inhibit the activity of main protease Mpro and reduce ACE-2 activity, the latter being the viral receptor of SARS-CoV-2 in humans. Alternately, naringenin may exert a therapeutic effect by ameliorating inflammatory responses, which is a hallmark of COVID-19 [110]. The inhibition potential of Mpro by naringenin has been shown through in silico analysis with the consequent inhibition of viral replication [111]. 

SARS-CoV-2 shares varying percentages of genetic homology with SARS-CoV (79.5%) and MERS-CoV (about 50%) [30]. Inhibition of the two-pore ionic channels TPC1 and TPC2 reportedly reduced MERS-CoV infectivity and viral replication [112,113,114]. It is possible that a similar result can be obtained with SARS-CoV-2 leading to reduction in viral replication [115]. It is interesting that naringenin can inhibit TPC1 and TPC2 in both humans and plants [116] and thus possesses the potential of inhibiting SARS-CoV-2 through this mechanism. In TPC2-silenced Huh7.5 cells (silenced with siRNA), infection with the coronavirus HCoV 229E was inhibited significantly compared to the control, which indicates that TPC2 plays an active role in coronavirus infection. Since naringenin can inhibit TPC2, it has been proposed that TPC2 can be the molecular target of the compound [117]. 

To summarize, some of the various possible mechanisms through which naringenin can exert an antiviral influence on SARS-CoV-2 include decreasing ACE2 expression in rat kidneys [118] and regulating release of cytokines such as TNFα and IL-6 from macrophages and T cells [119]. Additionally, naringenin has been shown to have an effect on CD4^+^ T cell proliferation and inhibit helper T cell (Th) 1 and 17 differentiation, both cells being proinflammatory and participates in the development of autoimmunity and tissue damage [120]. It is well-known that SARS-CoV-2 infection can lead to severe interstitial pneumonia through dysregulation of autoimmune and autoinflammatory responses [121]. Exacerbation of neutrophil activation is a hallmark of severe COVID-19 patients [122]; in the mouse model of acute respiratory distress syndrome (ARDS), naringenin has been shown to reduce neutrophil infiltration, which in turn can reduce the severity of ARDS [123].

Molecular docking studies with Molecular Virtual Docker showed that naringenin possesses a high affinity for binding to the spike (S) protein of SARS-CoV-2 and its human receptor hACE2 [124]. Overall, at least in the case of the Coronavirus SARS-CoV-2, the principle inhibitory actions of naringenin in humans can be mediated mainly in two ways. The first is through binding to Mpro, S protein, and/or the human receptor of the virus, hACE2. The second is through the suppression of the cytokine storm, which is the main cause of the severity of this viral disease leading to fatalities. Briefly, during COVID-19, there is an increase of proinflammatory cytokines and chemokines in the blood. These include interleukin-6 (IL-6), interferon-gamma (IFN-γ), monocyte chemoattractant protein-1 (MCP-1), inducible protein-10 (IP-10), interleukin-1β (IL-1β), tumor necrosis factor alpha (TNFα), granulocyte colony-stimulating factor (G-CSF), macrophage colony-stimulating factor (M-CSF), granulocyte-macrophage, colony-stimulating factor (GM-CSF), and macrophage inflammatory protein 1-α (MIP 1-α). This increase in proinflammatory cytokines such as IL-6, IL-1β, and TNFα results in what is known as a cytokine storm because their increase leads to the production of specific cytotoxic CD8+ T cells, which in turn stimulates antigen-specific B cells and antibody via CD4+ helper T cells. Hyperproduction of proinflammatory cytokines leads to edema and lung injury followed by other organ disorders [125,126,127].

Isoflavones such as daidzein (4′,7-Dihydroxyisoflavone) are members of the 7-hydroxyisoflavone group. Isoflavones may occur as aglycons (like daidzein) or glucosides as daidzin. This group of compounds occurs mainly in legumes, soy being the major food source for humans [128]. Various isoflavones have been shown to inhibit a variety of human and animal viruses, including adenoviruses, HSV, HIV, porcine Reproductive and Respiratory syndrome virus, and Rotavirus. The underlying inhibitory mechanisms of isoflavones include inhibition of virus binding to the host cell, entry, replication, and viral protein translation, among other modes of virus inhibition [129].

Daidzin is the 7-*O*-glucoside of daidzein. Although not as effective as kaempferol, daidzin demonstrated inhibition of Japanese encephalitis virus RNA replication [130]. It has been reported that in Madin–Darby canine kidney (MDCK) cells infected with Influenza A virus H1N1 (PR/8/34), daidzein caused a significant elevation of 5-hydroxyeicosatetraenoic acid, which was inhibited by the 5-lipoxygenase inhibitor, zileuton. It was further observed that virus replication was inhibited when cells were treated with 5-hydroperoxyeicosatetraenoic acid, a precursor of 5- hydroxyeicosatetraenoic acid and 5-lipoxygenase primary product. Taken together, the results suggest that daidzein regulated viral replication through products of 5-lipoxygenase [131].

Heat shock protein family A (Hsp70) member 5 (HSPA5), also known as binding immunoglobin protein (BIP) or glucose-regulated protein (GRP78) is an important cell surface receptor for facilitating viral entry into a number of viruses such as Japanese encephalitis virus and Coxsackie virus A9 [132,133]. HSPA5 acts as an alternate entry point for a number of human viruses, such as human Papilloma virus, Ebola virus, Zika virus, and human Coronaviruses (hCoVs) [134,135,136]. SARS-CoV-2 spike protein reportedly recognizes HSPA5 substrate-binding domain β (SBDβ); molecular docking and molecular dynamics simulation studies show that daidzein can bind strongly to HSPA5 SBDβ [137], thus acting as an inhibitor to SARS-CoV-2 entry into cells, a result confirmed with other phytoestrogens in the same study (note that daidzein is a phytoestrogen).

In the absence of efficacious drugs for most viral diseases, flavonoids present a potential source for such drugs or at least can provide a scaffold for the synthesis of novel drugs. A number of such flavonoids with antiviral activities are shown in Table 1.

**Figure 3:**
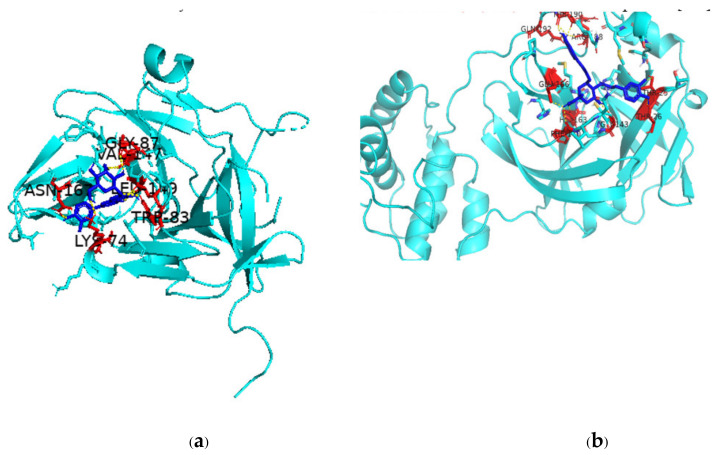
PyMol depiction of binding of flavonoids to DENV-2 NS2B-NS3 and SARS-CoV-2 Mpro (**a**) Binding of NS2B-NS3 activator-protein complex with luteolin-7-*O*-glucoside; (**b**) binding of rutin with Mpro. Protein is shown in cyan color, ligand as blue-colored stick structure, red-colored amino acids are involved in polar interactions with the ligand.

There are multiple ways in which flavonoids may inhibit any particular virus, but one major pathway is through inhibition of viral proteases, which play an integral role in viral replication. The binding of two such flavonoids to DENV NS2B-NS3 protease complex and SARS-CoV-2 Mpro active site is shown in Figure 3. The scientific literature is replete with studies on the inhibition of key viral proteases by flavonoids and so leading to inhibition of the virus itself [249,250,251,252]. In fact, a given flavonoid may inhibit different viruses in multiple ways. A recent review has concluded that the compound epigallocatechin gallate can inhibit Epstein–Barr virus by inhibiting replication; HIV by regulating oxygen level of target cells; arboviruses like Dengue, Chikungunya, West Nile virus, and Zika virus by suppressing infectivity, inhibiting viral RNA replication, and inactivating virus directly; Herpes simplex virus by inhibiting activity; Enterovirus 71 by suppressing viral RNA replication; and HBV by inhibiting viral promoter transcription [253]. Taken together, the available scientific reports suggest that the huge number of flavonoids that are present in various plant species can play a role as therapeutics against old and emerging viruses and that since quite a number of these flavonoids are dietary, they can make for easily available and affordable immune-boosters and anti-viral therapeutics [254].

### 3.2. Dietary Flavonoids

Flavonoids are widely present in leafy vegetables and fruits. Among dietary flavonoids, flavonols such as kaempferol, quercetin, myricetin, isorhamnetin, and rhamnetin and their glycosides such as rutin, isoquercitrin, and quercitrin are found in edible plant parts such as cranberry, apple, kale, lettuce, red pepper, onion, shallot, spinach, and broccoli. Flavanols are present in grapes, peaches, pears, mangoes, plums, and raspberries. Flavanones are present in citrus fruits. Anthocyanins are present in various berries [255]. The question then arises that if flavonoids are abundantly present in nature and humans can easily partake of them as vegetables and fruits, and if such flavonoids have antiviral properties, why are there viral infections and even pandemics?

### 3.3. Bioavailability of Flavonoids

Low bioavailability has always been a limiting factor for dietary flavonoids, despite the relative availability and consumption of dietary items containing flavonoid group of compounds [256]. The daily dietary intake of flavonoids has been estimated to be anywhere between 20 and 1000 mg [257]. To be absorbed, ingested flavonoids need to pass from the gut lumen into the circulatory system. Flavonoids are present in plants in the form of glycosides and these glycosidic moieties need to be removed before any absorption can take place. Hydrolysis of sugar moieties attached to the flavonoid and release of the aglycone is done by the enzyme lactase phlorizin hydrolase (LPH), found in the brush-border of the small intestine epithelial cells of mammals [258]. The aglycones then enter the bloodstream, but only after forming sulfates, glucuronides, and/or methylated metabolites, which are catalyzed by sulfotransferases, uridine-5′-diphosphate glucuronosyltransferases, and catechol-*O*-methyltransferases, respectively. Following this first phase of metabolism, after entering the bloodstream, flavonoid metabolites can undergo Phase 2 of metabolism in the liver prior to excretion in the urine [259].

### 3.4. Enhancement of Flavonoid Bioavailability through Various Means Other Than Nanotechnology

Since bioavailability is the determining factor of the in vivo bioactivity of flavonoids, numerous attempts have been and still are being made by scientists to increase bioavailability, even more so now that the importance of flavonoids in ameliorating various communicable and non-communicable diseases is being discovered and becoming widely known. The recognition of flavonoids as possible prophylactics and therapeutics has increased markedly since the first reports of their inhibitory effects against emerging, devastating pathogenic viruses such as Ebola [260] and currently COVID-19 [261] and further including currently not so widely known, but potentially fatal pandemic-causing viruses like Lassa virus (belonging to the Arenaviruses, which also includes Junin virus, Machupo virus, and Chapare virus) [262]. For increasing bioavailability of individual flavonoids, several methods have been tried (the use of nanotechnology for the effective delivery of flavonoids will be discussed in a later section).

The enhancement of intestinal absorption of daidzein has been reported with a eutectic mixture of borneol/menthol and microemulsion. The microemulsion formulation was prepared with ethyl oleate, Cremophor RH 40 (PEG40 hydrogenated castor oil), PEG400 (polyethylene glycol 400), and water. The mixture and microemulsion reportedly enhanced daidzein absorption in vitro [263]. Methylation of dietary flavones reportedly improves their intestinal absorption and stability in the liver [264]. Compared to unmethylated flavones such as chrysin and apigenin, methylated flavones such as 7-methoxyflavone or 7,4′-dimethoxyflavones improved intestinal absorption and greater metabolic stability [265]. Methylation may also protect dietary flavonoids from rapid hepatic metabolism, as shown in a comparative study between the non-methylated flavone galangin (3,5,7-trihydroxyflavone) and the methoxy flavones 5,7-dimethoxyflavone and 3′,4′-dimethoxyflavone [266]. The monomethylation of genistein and kaempferol increased their affinities for transport proteins such as human serum albumin and ovalbumin by 2–16 times compared to their non-methylated counterparts [267].

The bioavailability of hesperidin was increased by the removal of rhamnose group to yield the corresponding flavonoid glucoside (i.e., hesperetin-7-glucoside). The absorption site was also changed from the colon to the small intestine [268]. A double-blind, randomized, cross-over study in healthy human volunteers administered orange juice with the natural flavonoid naringenin-7-*O*-rutinoside or its α-rhamnosidase treated compound, naringenin-7-*O*-glucoside, showed that the latter had a 4-fold increase in the area under the plasma-time curve and the peak plasma concentration was 5.4-fold higher [269]. Other conventional methods suggested to increase the bioavailability of dietary flavonoids include modification of food preparation methods through the use of additives, modification of cooking process, and use of microorganisms, as in fermented foods [270]. It has been reported that cooking in water (a process that needs boiling) results in the loss of anthocyanins in berries [271], 70–85% loss of quercetin and kaempferol in broccoli [272], and decreased polyphenols in broccoli and white cabbage [273], so consumption of steamed fruits and leafy greens has been proposed as a solution [274].

Other methods have been experimented with to enhance the bioavailability of flavonoids. Plasma epigallocatechin concentrations were increased in mice administered orally a green tea (*Camellia sinensis*) extract along with steamed rice supplement [275]. The bioavailability of cocoa chocolate flavanols has been shown to be affected by the type of sugar associated with them; total flavanol absorption was higher with associated sucrose than with maltitol [276]. In a randomized, single-blinded, diet-controlled cross-over study, quercetin from quercetin-enriched cereal bars was found to be more bioavailable than quercetin powder-filled hard capsules [277]. Gut microbiota has been recognized to play a significant role in the bioavailability of flavonoids [278]. That would suggest that introducing beneficial microbes as probiotics may increase flavonoid bioavailability. Indeed, it has been shown that the probiotic *Lactobacillus paracasei* A221 improves the functionality and bioavailability of the kaempferol glucoside present in kale [279].

Taste plays a major role in individual selection of foods to eat. Thus, while some individuals might like raw or steamed leafy vegetables, others would want such vegetables to be cooked with the addition of salts and spices. What can also be said with certainty is that while fruits are more easily eaten raw (especially when ripe), that is not the case with leafy green vegetables. Therefore, the situation calls for finding other means to increase and improve the bioavailability of dietary flavonoid compounds to utilize these compounds’ usefulness both in maintaining a healthy body in general as well as during diseases. Scientists are increasingly turning to nanotechnology for efficient delivery of flavonoid compounds to target organs in a sustainable manner.

## 4. Utilizing Nanotechnology to Improve Delivery and Bioavailability of Flavonoids

Nanotechnology can solve the twin problematic issues with flavonoids, namely, low water solubility, which plays the major role in low bioavailability. A typical example can be taken as fisetin, a hydrophobic dietary flavone (present in strawberries, apples, cucumbers, and onions), whose low aqueous solubility (less than one mg per mL) and resultant low bioavailability limits its use despite its many health benefits and potential as an anti-COVID-19 therapeutic [280,281]. To make steps towards making available the manifold biopharmaceutical properties of fisetin, it is necessary to improve its bioavailability. A number of nanotechnology applications have been proposed to that effect, considering the lipophilic nature of fisetin, which includes nanoemulsion, liposomes, and nanoparticles [282,283,284]. To improve the bioavailability of dietary flavonoids, Ninfali and others in their review [186] mentioned micro/nanotechnogy delivery strategies such as micelles, nanoparticles, microspheres, crystals, dendrimers, the Self-Micro-emulsifying Drug Delivery System (SMDDS), and the Self-Nanoemulsifying Drug Delivery systems (SNEDDS) (also reviewed by other authors [285,286]).

### 4.1. Micelles

Polymeric micelles can be described as self-aggregating colloids made by amphiphilic polymers. Above the critical micellar concentration (CMC), individual molecules will self-assemble forming an inner hydrophobic core with an external hydrophilic shell.

Their small size (10–100 nm) allow them to reach difficult target organs, the hydrophobic nature of the core allows for the solubilization of hydrophobic compounds (like flavonoids), and the hydrophilic shell enables them to prolong their blood circulation time [287]. The application of micelles for delivery of a poorly aqueous soluble flavonoid like quercetin will be discussed in some detail to provide an understanding of use of micelles to deliver poorly soluble and not very stable flavonoids to target cells or organs.

In vitro evaluation of quercetin-encapsulated polymeric micelles were carried out with methoxy polyethylene glycol-*b*-poly(d,l-lactide) (mPEG-PLA) and methoxy polyethylene glycol-*b*-poly(ε-caprolactone) (mPEG-PCL) copolymers as a drug delivery platform for quercetin. The copolymers were synthesized having different molecular weights of the hydrophobic blocks to investigate weight and polymer type variations on the micelle properties; mPEG5K-PLA3K had the highest compatibility with quercetin according to the Flory–Huggins interaction parameter (a parameter defining thermodynamic properties of polymer solutions) and showed highest loading and encapsulation for quercetin. The release of the compound was biphasic and was clearly dependent on drug–copolymer compatibility. Overall, the experiment showed that micelles can act as carriers for flavonoid compounds [288]. 

Encapsulation of myricetin (3,5,7-Trihydroxy-2-(3,4,5-trihydroxyphenyl)-4H-chromen-4-one) in polyvinyl caprolactam–polyvinyl acetate–polyethylene glycol graft copolymer (PVCL-PVA-PEG) polymeric micelles has been found to increase its aqueous solubility and stability, as well as corneal permeability. The results indicated that, in this form, myricetin can be used for clinical applications in ophthalmology [289]. Apigenin-loaded polymeric micelles composed of Pluronic P123 and Solutol HS 15 with a diameter of 16.9 nm showed sustained release of apigenin as well as greater cytotoxicity against HepG2 and MCF-7 cancer cells in vitro than that of the free compound [290].

### 4.2. Nanoparticles

Nanoparticles are colloidal particles less than 1 μM in diameter. They can be made as nanospheres or nanocapsules. Depending on the materials used for their manufacture, they can be further classified into inorganic and organic nanoparticles. Inorganic or metallic nanoparticles reportedly show inherent anti-viral activity [291]. Gold and silver nanoparticles (NPs) form the dominant ones used; these two NPs along with superparamagnetic iron oxide NPs have formed the major NPs to be used against HIV [292,293,294]. Silver NPs have shown antiviral activity against a number of viruses including HIV, HSV, HBV, respiratory syncytial virus, monkey Pox virus, and human Metapneumovirus (HPV) [295,296]. It has been shown that silver nanoparticles using fungi can be synthesized and these NPs can inhibit HSV types 1 and 2, and human Parainfluenza virus type 3 in a size-dependent manner. The NPs can reduce virus infectivity, which has been hypothesized to occur due to blocking of cell–virus interactions [297]. Silver NPs also reportedly inhibited Peste des petits ruminants virus replication [298].

Silver nanoparticles have been tested with success against influenza viruses. Xiang and others [299] studied the effect of silver NPs on H3N2 virus and found that the NPs can destroy the morphological structure of the virus within a short period of time from 30 min to 2 hours. The efficacy of silver NSPs against Influenza virus H3N2 was further shown by Miao and his group [300]; similar results were obtained with H1N1 [301]. In another study, montmorillonite clay-based nana silica platelets (NSPs) surface modified with silver NPs showed antiviral activity against Influenza virus A, as well as other viruses such as Japanese encephalitis virus and DENV [302]. In fact, the use of NPs against Influenza virus encompasses not only use of NP itself as an anti-viral agent or the use of NPs for drug delivery, but also the use of NPs as immunity-inducing vaccines and the use of NPs in gene silencing approaches [303]. Monodisperse silver nanoparticles have been reported to inhibit HBV replication [304]. Silver NPs reportedly can inhibit Tacaribe virus if administered prior to virus infection or at an early phase after viral exposure [305]. Taken together, silver (Ag) NPs can inhibit a wide variety of viruses; the list includes HBV, Influenza virus, and various strains of HIV-1; however, AgNPs were cytotoxic at concentrations above 6 μg/mL [306].

Other metal oxide NPs have shown potential for being anti-viral therapeutics; zinc oxide (ZnO) NPs and polyethylene glycol (PEG)-coated ZnO-NPs have been shown to be inhibitory to both HSV-1 and Influenza A/Puerto Rico/8/34 (H1N1; PR8) viruses [307,308]. Silver and copper oxide NPs showed, respectively, strong and moderate anti-viral activity against SARS-CoV-2 virus [309]. Titanium dioxide NP by itself, and in complex with another metal oxide NP is now recognized as an effective anti-viral tool. Titanium dioxide (TiO_2_) NPs have been shown to possess virucidal effect against Influenza virus in suspension or when absorbed on a film [310]. Faba bean plants treated with TiO_2_ NPs showed a significant reduction in disease severity caused by the broad bean stain virus (BBSV), leading to enhanced growth of TiO_2_-NP-treated plants [311]. TiO_2_-NPs exhibited strong anti-SARS-CoV-2 activity at very low cytotoxic concentrations, leading to the recommendation that these NPs may be used in vitro and in wall coatings to serve as a potent disinfectant of the Coronavirus [312]. An efficient visible light-sensitive photocatalyst has been described containing copper oxide nanoclusters grafted titanium dioxide (Cu_x_O/TiO_2_). The Cu_x_O nanocluster comprises of both Cu(I) and Cu(II); the former denaturalizes viral proteins even under dark conditions. Under visible light, Cu(II) is converted to Cu(I) and photogenerated holes in the valence band of TiO_2_ lead to strong oxidation power leading to the oxidation of virus surface proteins and consequent virus destruction [313].

It is well established that metallic nanoparticles can play an inhibitory role on a number of viruses. However, efforts are underway to increase the efficacy of metallic NPs with organic compounds. A case in point is the use of tannic acid modified silver NPs, which reduced HSV-2 the strain 333 infectivity both in vitro and in vivo compared to silver NPs by themselves. Since tannic acid has a high affinity for proteins and sugars, it has been hypothesized that incorporated tannic acid in the modified silver NPs would bind to the virion glycoproteins, making them inert with subsequent loss of attachment and entry ability to host cells [314].

Scientists are taking the issue of anti-viral nanoparticles a step further with the synthesis of ‘green nanoparticles’ (GNPs), which mainly combines silver nanoparticles (SNPs) because of the higher antiviral efficacy of silver with plant products, the latter comprising of single or multiple phytochemicals or crude plant extracts. Haggag and others used aqueous and hexane extracts of the plants *Lampranthus coccineus* (Haw.) N.E.Br. (Aizoaceae) and *Malephora lutea* (Haw.) Schwantes (Aizoaceae) to synthesize green SNPs. The spherical nanoparticles produced from the two plants ranged from 10.12 nm to 27.89 nm and 8.91 nm to 14.48 nm for the aqueous and hexane extracts of the two plants, respectively. The green SNPs reportedly exhibited remarkable anti-viral activity against Herpes simplex virus 1 (HSV-1), Hepatitis A virus strain 10 (HAV-10), and Coxsackie B4 virus. Molecular docking studies were in agreement with the results obtained with green SNPs from the two plants; various phytochemicals exhibited high docking affinities for HSV-1 thymidine kinase, HAV-10 3c proteinase, and Coxsackie B4 virus protease [315].

Green silver NPs prepared with silver nitrate and methanolic extracts of strawberry (*Fragaria ananassa* Duch.) or ginger (*Zingiber officinale* Roscoe) have been reported for their strong anti-viral activity against SARS-CoV-2, a finding in agreement with binding of the common phytochemical neohesperidin with both human AAK1 (AP2 associated kinase 1) protein and SARS-CoV-2 NSP16 protein [316]. Green silver NPs prepared with silver nitrate and *Curcuma longa* L. (Zingiberaceae) tuber powder extracts have been mentioned as being of potential use for biomedical purposes [317]; notably, curcumin, a polyphenolic phytochemical ((1,7-bis(4-hydroxy-3-methoxyphenyl)-1,6-heptadiene-3,5-dione), also called diferuloylmethane) found in *Curcuma longa* tubers, is regarded as a potential treatment for COVID-19 [318]. The antiviral properties of curcumin have been reviewed and the compound reportedly has antiviral effects against RNA viruses such as HIV, Zika virus, DENV, CHIKV, vesicular stomatitis, Influenza A, Enterovirus 71, human Respiratory syncytial virus, Norovirus, viral hemorrhagic septicemia virus, porcine reproductive and Respiratory syndrome virus, transmissible gastroenteritis virus, and the Coronaviruses SARS and SARS-CoV-2; the compound is also active against DNA viruses such as HSV-2, Kaposi’s sarcoma-associated Herpes virus, bovine Herpesvirus 1, and human Adenovirus [319].

Silver nanoparticles (AgNPs) have become possibly the strongest candidates as anti-viral agents. Overall, their action can be two-fold: interact with the outer coat of a virus and so prevent viral attachment to its cellular receptor or interact with viral DNA or RNA and so inhibit replication and propagation within the host cell [320]. Magnetic hybrid colloid (MHC), which has been decorated with AgNPs of varying sizes, has proven effective in inactivating and reducing bacteriophage ϕX174 and murine norovirus (MNV) [321]. It has been suggested that AgNPs inhibit HIV-1 through binding to gp120 (gp120 plays a vital role in the ability of HIV-1 to enter CD4+ cells) and so preventing the CD-4 dependent binding, fusion, and infectivity of the virus [322]. AgNP/chitosan (Ch) composites reportedly showed AgNP concentration-dependent inhibitory effect against H1N1 Influenza A virus [323]. However, the mechanism of inhibition of the virus by AgNP/Ch composite remains to be elucidated.

In addition to the metallic nanoparticles, use has also been made of calcium phosphate NPs, but their use has mainly been limited to therapeutic applications in bone regeneration. Their other uses include delivery of antibiotics and anti-inflammatory drugs to bones [324]. Another review has discussed applications of calcium phosphate NPs in gene silencing and transfection [325].

Nanoparticles have been described as ‘nanodrones’. It has been hypothesized that hACE-2 can be targeted with such nanodrones containing flavonoids as a tool to combat COVID-19, where the flavonoids are encapsulated within the nanodrones using microfluidic approaches. Such an approach has been shown to be fruitful for enabling higher amounts of ‘payload’ to reach lungs with lesions; a point to remember is that lungs are the primary site of SARS-CoV-2 infections and subsequent damages [326,327]. However, to our knowledge, any such nanodrones are yet to be tried against any viral targets including SARS-CoV-2. 

A number of antiviral flavonoids such as quercetin, fisetin, rutin, and myricetin, as well as other polyphenols with anti-viral potentials against viruses such as Ebola, Polio, HCV, Coronavirus (SARS-CoV), Cytomegalovirus, HSV 1 and 2, and Respiratory syncytial virus, as well as having brain boosting effects, have been proposed to be nanoencapsulated and used against COVID-19 to give dual effects of anti-viral and brain boosting. For encapsulation, metallic nanoparticles including superparamagnetic iron oxide nanoparticles (SPION) have been tried for quercetin, but this line of approach (SPION) despite its advantages (superparamagnetic nature, high ratio of spin polarization, and elevated conductivity) has also failed due to metallic toxicity [328]. An interesting application of the flavonoid naringin was to use it in the chalcone form, which was then surface-absorbed to gold, silver, or lead nanoparticles for the extraction of zein; however, the method is yet to yield antiviral uses [329].

Organic and inorganic nanocarrier (NC) systems are being developed for synthetic antiviral drugs to enhance drug availability, dampen drug degradation, and improve cell-targeted drug delivery. Organic NCs include liposomes, dendrimers, carbon nanotubes, and micelles, while inorganic NCs consist of various metallic NPs [330]. Interestingly, synthetic anti-viral drugs and most flavonoids suffer from the same problems of poor aqueous solubility and reduced bioavailability, but while intense efforts are going on for synthetic drugs, such efforts seem to be lacking for bioactive drugs like flavonoids. However, interest is picking up on flavonoids but for a different purpose. Since NPs greater than 6 nm are excreted through the renal excretion system with difficulty, they accumulate in organs causing oxidative stress and toxicity. The use of anti-oxidants such as flavonoids are being considered to reduce this oxidative stress when co-administered with synthetic drugs [331]. This approach can open up a novel integrated combination of synthetic and antiviral flavonoid drug delivery for obtaining better treatment results.

To conclude this section, silver nanoparticles appear to be the most effective antiviral agents compared to other metals. These nanoparticles can be used by themselves or in combination with plant materials directed against specific viruses or vectors. The issue of silver nanoparticles (AgNPs) containing plant extracts or plant components for anti-cancer and anti-viral uses has been reviewed extensively by Jain and others [332]. Some of the plant materials used in various studies are mentioned with the viruses/vectors they have been used for, given in parenthesis. Amongst others, AgNPs containing *Aquilaria sinensis* essential oil (Dengue and Zika vectors), *Pogostemon cablin* essential oil (Dengue and Zika vectors), combination of *Andrographis paniculata*, *Phyllanthus niruri*, and *Tinospora cordifolia* (Chikungunya virus), *Cinnamomum cassia* (H7N3 Influenza A virus), and *Moringa oleifera* seed extract (DENV2) have been reportedly evaluated for their anti-viral or anti-vector (like mosquito larvicidal) potential [333,334,335,336].

The use of single flavonoids, like quercetin, in different types of nanocarriers has been studied against various types of cancer but not against viruses. Xu and others reported that quercetin containing monomethoxy poly(ethylene glycol)-poly(ε-caprolactone) nanomicelles demonstrated induction of cellular apoptosis and inhibited cell proliferation and tumor angiogenesis in colorectal cancer [337]. Guan and others showed that poly (lactic-*co*-glycolic acid)-d-α-tocopheryl polyethylene glycol 1000 succinate nanoparticles showed more effective encapsulation of quercetin and better targeting of malignant neoplastic cells [338]. Quercetin-containing amphiphilic chitosan nanoparticles were shown to give higher flavonoid release at acidic pH in breast cancer [339]. However, none of the above studies involved viruses; on the other hand, such studies may pave the way for delivering flavonoids to target tissue(s) utilizing nanotechnology techniques.

### 4.3. Self-Nanoemulsifying Drug Delivery Systems (SNEDDS) and Other Nano-Delivery Systems

Nanoemulsions have been defined as the mixture of two immiscible liquids like water in oil (W/O) or oil in water (O/W) where the liquids are heterogeneously dispersed, forming droplets sizes in the range of 20–200 nm [340]. The O/W nanoemulsions are more suitable for the delivery of hydrophobic drugs, the W/O nanoemulsions are more suitable for the delivery of hydrophilic drugs. These characteristics allow nanoemulsions to deliver flavonoids as well as other biomedicines, which may be hydrophobic or hydrophilic in nature. Nanoemulsions can highly improve the oral bioavailability, as demonstrated in the case of curcumin [341].

The components of SNEDDS may vary with the type of drug used for delivery. For instance, the bioavailability of zedoary turmeric oil (oil from dry rhizomes of *Curcuma zedoaria* Rosc., Zingiberaceae family) was improved with the presence of Transcutol (diethylene glycol monoethyl ether), ethyl oleate, and Tween 80 [342]. On the other hand, the oral delivery of the hepatoprotective phytochemical oleanolic acid was improved in the presence of Cremophor EL, Labrasol^®^, Transcutol, Sefsol^®^ 218, Cremophor RH40, and PG (propylene glycol) [343]. The natural antioxidant isoflavone found in soy-derived foods and a proposed nutraceutical for COVID-19 [344] was seen to be 100% released within 5 min when SNEDDS contained Labrafac Lipophile^TM^ 1349, Maisine 35, Transcutol, Cremophor EL, and Labrasol [345]. P-glycoprotein (P-gp) is a multidrug resistance efflux pump present in various body areas (including the gastrointestinal or GI tract) and prevents the entry of drugs in the systemic circulation. The use of surfactants such as Cremophor EL, Labrasol, or Solutol^®^ HS15 is necessary to inhibit P-gp efflux [346,347]. 

Coenzyme Q10 (CoQ10) is present in many foods that we eat, is hydrophobic in nature, and is also an antioxidant. A SNEDD formulation has been designed to increase the oral bioavailability of the compound. The optimal formulation contained CoQ10, Witepsol H35, Solutol HS15, and Lauroglycol FCC in a weight ratio of 1:0.7:4:2 [348]. A SNEDD formulation of buckwheat flavonoids containing, in addition, PEG-40 hydrogenated castor oil, propylene glycol, and castor oil showed that the formulation was a promising oral delivery system with improved potential of bioavailability [349]. The notable thing about this finding was that both common buckwheat (*Fagopyrum esculentum* Moench, Polygonaceae family) and tartary buckwheat (*Fagopyrum tataricum* (L.) Gaertn.) possess a diverse array of bioactive phytochemicals, which have anti-oxidant and anti-cancer properties [350].

Naringenin (4′,5,7-trihydroxyflavanone), which is widely found in grapefruit and other citrus fruits [351], is reported to have beneficial effects such as anti-oxidant, anti-inflammatory, anti-cancer, and anti-atherogenic [352,353,354,355]. However, its poor aqueous solubility and low bioavailability following oral administration are major limitations behind the compound’s use as a therapeutic agent. Khan and others have reported that the use of Triacetin as an oil, Tween 80 or Cremophor EL as a surfactant, and co-surfactant Transcutol HP synthesized SNEDDS containing naringenin resulted in higher naringenin release and absorption compared to naringenin alone in in vitro studies [356].

Nanoemulsion containing flavonoid-rich extract of *Achyrocline satureioides* (Lam.) DC. showed higher inhibitory effects on HSV-1 replication than extract or pure quercetin alone. The results suggested that this nanoemulsion containing plant extract can be used for topical application in Herpes infections [357]. It has also been shown that *Carica papaya* L. leaf extract silver synthesized nanoparticles gave promising inhibitory effects in vitro against DENV-2 with IC_50_ value of 9.20 μg/mL [358]. Nanoparticles of pomegranate peel extracts reportedly showed inhibitory activity against Tobacco mosaic virus (TMV) [359]. Various nanoformulation components and stabilizers have been tested for improving the solubility of various flavonoids in nanoemulsions, as reviewed by Zhao and co-authors [360]. The various stabilizers for nanoemulsions include d-α-tocopherol polyethylene glycol 1000 succinate (TPGS) for naringenin and myricetin; Poloxamer 188 (PXM 188) for scutellarin and kaempferol; the various components for nanoemulsion of baicalin include Isopropyl myristate (IPM, Polyoxyethylene castor oil (Cremophor EL 35) and Polyethylene glycol 400 (PEG 400) [361,362,363,364]. *Artemisia rupestris* L. flavonoid nano-encapsulation using polylactic-co-glycolic acid (PLGA) showed an enhanced anti-HBV effect in vitro compared to the flavonoid original extract without nano-encapsulation [365]. However, quercetin nanoparticles prepared by nanoprecipitation technique (without any nano-encapsulation) demonstrated a significant inhibitory effect on avian Influenza virus H5N1 strain (EPI573317) as indicated by reduction of virus titer and cytopathogenic effect (CPE) in MDCK cells [366].

## 5. Discussion

Several things appear to be beyond dispute in the quest for nanotechnological applications of flavonoids against viral diseases of different types and sorts. The first is that various viruses have been present long before the emergence of the current zoonotic viruses SARS, MERS, or SARS-CoV-2 or other viruses like West Nile virus, Nipah, Hanta, Hendra and a host of other zoonotic and non-zoonotic viruses [367]. The second is that the plant kingdom contains a huge number of flavonoid compounds (possibly 6,000 or more divided into several groups and subgroups) [66], and dietary foods like leafy greens and fruits are good sources of such flavonoids [255]. The third is that flavonoids (flavonoid aglycones) have very little solubility in water [368], are destroyed during cooking, and even when taken orally in the raw state (as in fruits), have limited bioavailability because of their poor aqueous solubility. The fourth is that a vast number of in silico studies and a number of in vitro and in vivo studies have shown that various dietary flavonoids have strong anti-viral potential, in fact possibly more than existing anti-viral synthetic drugs; also to be considered is the latter’s (synthetic drugs) limited efficacy and serious adverse effects [369], but with less or no toxicity but more affordability for flavonoids. The whole scenario now calls for delivery of flavonoids in a manner with increased bioavailability.

Nanotechnology, possibly rightly so, has become the latest buzz word among scientists to solve many if not all problems. This also applies to diagnosis of viral diseases, disinfection of homes and surfaces from virus (and other pathogens), and application of various synthetic and natural compounds as prophylactics or therapeutics against different viruses. Flavonoids, theoretically, in combination with nanotechnology, should have been instrumental in solving the present pandemic caused by the coronavirus SARS-CoV-2 a long time ago because of the manifold techniques of nanotechnology, which include the various nanocarriers capable of delivering both lipophilic and hydrophilic compounds [370]. At the same time, flavonoid compounds with their extensive diversities in nature, and also acting as scaffolds for addition of other chemical moieties, could have made potential therapeutic for any given virus. Although the present manuscript can be described as a scoping review, there are enough evidence to suggest that flavonoids, singly or in combination with other flavonoids, synthetic drugs or even metal nanocarriers can potentially inhibit any old or emerging virus. However, in practice, this has not been the case, most exemplified by the SARS-CoV-2 pandemic. Even with numerous in silico studies demonstrating the binding of various flavonoids to integral viral proteins or the human ACE-2 receptor of the SARS-CoV-2 virus, and another batch of studies promoting the effectiveness of various nanodelivery systems, thus far there has been not a single discovery of a flavonoid or for that matter any other compound and a nanodelivery system as an anti-SARS-CoV-2 drug with an efficacious delivery system. To quote a recent review, “well-designed high-quality studies are needed to further study the effectiveness and safety of these potential drugs in order to provide valid recommendations for better control of the COVID-19 pandemic” [371]. 

Despite the effort by scientists, there are still a number of problems, including toxicity and environmental concerns, to be solved; some of them regarding metallic NPs have been very succinctly mentioned by Mori and others [323]. For instance, metallic nanoparticles can end up in other places such as waterbodies. Ag-NPs can not only cause cytotoxicity and genotoxicity in fish cells of Japanese medaka (*Oryzias latipes*) [372] but can also inhibit algal (*Chlamydomonas reinhardtii*) photosynthesis [373]. Ag-NPs reportedly showed disruption of glial cell line-derived neurotrophic factor (GDNF)/Fyn kinase signaling in spermatogonial stem cells [374]. On the other hand, zinc nanoparticles were shown to ameliorate Ag-NPs-induced adversely affected sperm motility morphology, viability, and concentration in adult male rats [375].

SNEDDS formulation containing *Nigella sativa* L. seed oil demonstrated toxicity against adult zebra fish (*Danio rerio*) with a LC_50_ of 154.637 ± 75.609 ppm [376]. In sub-chronic toxicity studies, SNEDDS containing ethyl acetate extract of bay leaf (*Eugenia polyantha* Wight) showed, in rats, minor damage of liver and kidneys and moderate damage of pancreas [377]. Any possible toxicities arising from excipients, surfactants, co-surfactants, and oils, as well as the main drug material, needs to be taken into consideration not only for SNEDDS, but also for other nanoformulations.

To summarize, even as this has been said before, flavonoids hold a strong promise for the cure of viral diseases, and various nanodelivery systems and nanoformulations can play an important role in the delivery of lipophilic flavonoids, which can ensure targeted drug delivery and increased bioavailability of the flavonoid compounds. Once the whole process has been perfected, this may change our present concepts of various antiviral drugs and their delivery methods. However, at present, we are still waiting for the big breakthrough — a combination of targeted delivery of flavonoid(s) utilizing one or other form of nanotechnological means, as both prophylactic and therapeutic uses for old and emerging viruses.

## 6. Materials and Methods

The nature of this review made the search of the various materials conducted under separate headings, but with the broad theme of the review in mind. Specifically, the three key combinations were <nanotechnology, delivery systems>, <flavonoids, anti-viral or types or bioavailability or hydrophobicity>, and <nanotechnology delivery systems, flavonoids>. In addition to these three main search combinations, the complex nature of the review necessitated individual searches such as <naringenin, anti-viral>, <phloretin, antiviral>, and similar searches. Searches were conducted in PubMed, PubMed Central, and Google without any set time limits. However, the sheer number of papers compelled the authors to go through the titles and abstracts of what seemed like relevant papers and then search the reference sections of relevant papers for additional papers, a method which was more promising and resulted in less searching through what turned out to be redundant or duplicate papers after reading them. The complexity of the search can be given by a few examples of numbers of papers versus some of the selected search terms. Thus, searching through PubMed Central, <nanoparticle application of flavonoids, viral diseases> gave 1396 hits; <flavonoids, nanotechnology, anti-viral> gave 555 hits; <flavone, anti-viral> gave 1924 hits; <flavonol, anti-viral> gave 2943 hits; <daidzein, anti-viral> gave 624 hits; <isoorientin, virus> gave 165 hits; and <flavonoids, anti-virals, review> gave 6690 hits, just to give a few examples. However, each selected paper was read by at least two authors before any contents of the paper were deemed suitable for referencing, citing, or discussing in the present review.

## 7. Conclusions

Despite progresses made, more studies are necessary to translate the potential of nanotechnology to deliver hydrophobic anti-viral flavonoid drugs of low bioavailability to target areas maintaining increased bioavailability and sustained release ability. 

## Figures and Tables

**Table 1:** Some promising anti-viral flavonoids.

Flavonoid	Antiviral Activity ^1^ and Reference
Axillarin	Rhinovirus type 2 [138]
Myricetin	Moloney murine leukemia virus [139], SARS-CoV [140], influenza viruses [141], HIV-1 [74], Rauscher murine leukemia virus [142], African swine fever virus [67], Zika virus [70], Enterovirus A71 (EV71) [71], SARS-CoV-2 [79], Feline calicivirus (FCV-F9) [143], Murine norovirus [144]
Myricetin-3-rhamnoside	HIV-1 [76]
Myricetin-3-*O*-rhamnoside	Hepatitis B virus (HBV) [73], Influenza A virus (H1N1) [77], HIV-1 [76]
Myricetin-3-*O*-rutinoside	SARS-CoV-2 [81]
Chrysoeriol	Rhinovirus type 2 [138]
Chrysoeriol- 6-*C*-β-D-boivinopyranosyl-4′-*O*-β-D-glucopyranoside	Hepatitis B virus [145]
Diosmetin	Rhinovirus type 2 [138], Hepatitis C virus HCV) [146], Enterovirus A71 (EV-A71) [147]
Diosmin	SARS-CoV-2 [148]
Hesperidin	SARS-CoV-2 [148], Zika and Chikungunya virus (CHIKV) [149], Influenza A virus [150]
Hesperetin	CHIKV [151], Yellow fever virus [152], HSV-1 [153], Sindbis virus [154]
Bicalein	Human cytomegalovirus (HCMV) [155,156], Avian influenza virus H5N1 [157], Influenza virus H1N1 [158], Sendai virus [159], DENV-2 [160,161,162]
Baicalin (baicalein-7-glucuronide)	CHIKV [100], Influenza virus H1N1 [158], Sendai virus [159], DENV-2 [160,161,162], EV-A71 BrCr-Tr strain [163], HIV-1 [164]
Chrysin	EV-A71 [165], Influenza virus A/Puerto Rico/8/34 (A/PR/8) [166], DENV and Zika virus [167] ^2^, Coxsackie virus B3 ^3^ [168]
Fisetin	DENV-2 [102,103], CHIKV [169]
Isorhamnetin	Rhinovirus type 2 [138], Influenza A H1N1 virus [170], SARS-CoV-2 [171], HCV [172]
Chrysosplenol C	Rhinovirus type 2 [138], Picornaviruses [173]
Luteolin	Japanese encephalitis virus (JEV) [174], HIV-1 [175], EV71 and Coxsackie virus A16 [176], SARS-CoV [177], DENV [178], Respiratory syncytial virus (RSV) [179], Influenza A virus (IAV)—subtypes A/Jiangxi/312/2006 (H3N2) and A/Fort Monmouth/1/1947 (H1N1) [180], CHIKV [181]
Vitexin	Parainfluenza type 3 (Para 3) [182], Herpes simplex virus (HSV) type 1 and Hepatitis A virus (HAV)—H10 [183], SARS-CoV-2 [184,185,186,187], Influenza A viruses, A/Puerto Rico/8/1934 (PR8-H1N1) and A/Chicken/Taiwan/3937/2012 (3937-H6N1) [188]
Isovitexin	Influenza A viruses, A/Puerto Rico/8/1934 (PR8-H1N1) and A/Chicken/Taiwan/3937/2012 (3937-H6N1) [188], Hepatitis B virus (HBV) [189,190], Respiratory syncytial virus (RSV) and Influenza A1 virus (FM1) [191]
Orientin	SARS-CoV-2 [192], Parainfluenza type 3 virus [193], Herpes simplex virus 2 (HSV-2) [194] ^4^
Isoorientin	Respiratory Syncytial virus (RSV) [195], SARS-CoV-2 [196,197]
Rutin ^5^	SARS-CoV-2 [198,199,200,201], Norovirus [202], DENV-2 [102,203], Vesicular stomatitis virus [204], Canine distemper virus [205], avian Influenza strain H5N1 [206], HCV [207]
Cyanidin	Anti-viral activity against various viruses of Cyanidin-3-rutinoside, Cyanidin-3-xylosylrutinoside, Cya-nidin-3,4′-di-O-β-glucopyranoside, Cyanidin-4′-O-β-glucoside, Cyanidin-3-O-glucoside, Cya-nidin 3-O-arabinoside, and cyanidin-3,5-O-diglucoside [208]; Influenza viruses A and B anti-viral activity with 3-*O*-α-l-rhamnopyranosyl-β-d-glucopyranosyl-cyanidin and 3-*O*-β-d-glucopyranosyl-cyanidin [209]; SARS-CoV-2 anti-viral activity with cyaniding 3-glucoside [210]
Kaempferol	Pseudorabies virus (PRV) [211], SARS (kaempferol, kaempferol glycosides, and acylated kaempferol glucoside derivatives (such as afzelin, juglanin, tiliroside, and tiliroside derivatives)) [212], Japanese encephalitis virus (JEV) [130,213] ^6^, Equine Arteritis virus (EAV) [214], SARS-CoV-2 [215,216,217,218,219], HIV-1 [220,221,222], H1N1 Influenza virus [223], HSV-1 and HSV-2 [224], H9N2 swine Influenza virus [225]
Epigallocatechin gallate	Chikungunya virus [226], HBV [227,228,229], HSV [230,231], Epstein-Barr virus (EBV) [232,233], HIV [234,235,236,237,238], HCV [239,240,241], Influenza virus [242], SARS-CoV-2 [243,244,245], Coxsackie virus B3 [246], EV71 [247], West Nile virus, DENV, Zika virus [90], Zika virus (ZIKV) [248]

^1^ Based on in vivo, in vitro, and in silico studies and to be noted that this is not a comprehensive list of all anti-viral flavonoids; ^2^ synthetic halogenated chrysin derivatives [167]; ^3^ several synthetic 4-substituted benzyl derivatives of chrysin were more potent than chrysin against Coxsackie virus B3 [168]; ^4^ mixture of orientin, rutin, quercetin, and kaempferol [194]; ^5^ rutin is also known as sophorin, rutoside, and quercetin-3-rutinoside; ^6^ kaempferol enhanced dengue infection in a GRP78-dependent manner [213].

## Data Availability

Not applicable.
